# Myocardial work in Chagas disease: comparative analysis with conventional parameters in different cardiac clinical stages

**DOI:** 10.3389/fcvm.2025.1688931

**Published:** 2025-11-03

**Authors:** Alex dos Santos Felix, Daniel Arthur Barata Kasal, Rodolfo de Paula Lustosa, Marcelo Imbroinise Bittencourt, Pedro Pimenta de Mello Spineti, Bruno Reznik Wajsbrot, Marcia Bueno Castier, Ricardo Mourilhe-Rocha

**Affiliations:** ^1^ Cardiology Department, Universidade do Estado do Rio de Janeiro, Rio de Janeiro, Brazil; ^2^ Echocardiography Department, Instituto Nacional de Cardiologia, Rio de Janeiro, Brazil; ^3^ Cardiology Department, Complexo Hospitalar Americas, Rio de Janeiro, Brazil; ^4^ DASA, Rio de Janeiro, Brazil

**Keywords:** Chagas cardiomyopathy, heart failure, myocardial work, cardiac imaging, strain echocardiography

## Abstract

Chagas disease (CD) is a neglected chronic infectious disease and represents an important cause of morbidity and mortality in endemic areas of Latin America. Chronic Chagas cardiomyopathy (CCC) is the most severe manifestation of the disease and the leading cause of death in these patients. Myocardial deformation, measured by speckle-tracking echocardiography (STE), has been proven to detect subclinical disease and has prognostic value in CCC, but it is load dependent. We aimed to characterize myocardial work (MW) as new, less load-dependent, echocardiographic variables in patients with different clinical stages of CCC and to assess its correlation with left ventricular (LV) ejection fraction (EF) and accuracy to detect LV dysfunction, compared with routinely used parameters. Fifty consecutive patients with CD were included. Clinical assessment, NT-proBNP, and a comprehensive 2D echocardiogram were performed. Patients were divided into four groups, depending on disease stages: Stage A—indeterminate form (*n* = 9), defined as asymptomatic patients without abnormalities on physical examination, electrocardiogram, or echocardiography; Stage B1, chronic Chagas cardiomyopathy (CCC) with preserved ejection fraction (*n* = 18); Stage B2, CCC with left ventricular global dysfunction (LVEF <55%, *n* = 13); and Stage C, CCC with LVEF <55% and clinical heart failure (*n* = 10). Patients in Groups B2 and C had lower values of global work index (GWI), global constructive work (GCW), and global work efficiency (GWE) than those in patients in Groups A and B1 (*p* < 0.001). Values of GWI, GCW, and GWE in Groups A and B1 were normal compared with normative data. Among the studied parameters, GCW showed the strongest correlation with LVEF (*r* = 0.854), followed by GWI (*r* = 0.848) and global longitudinal strain (GLS) (*r* = 0.810), while moderate correlations were observed for GWE (*r* = 0.578) and NT-proBNP (*r* = 0.634). Pairwise comparisons of correlation coefficients using Steiger's *Z* test revealed that GCW had a significantly stronger correlation with LVEF than NT-proBNP (*p* < 0.001), but no significant difference was observed when compared with GLS or GWI (*p* > 0.05). For the detection of LV global dysfunction as a categorical variable, defined as LVEF <55%, the best accuracy was observed for GCW (AUC = 0.976; 95% CI: 0.927–1.000; optimal cutoff 1,699 mmHg%), followed by GWI (AUC = 0.965; 95% CI: 0.905–1.000; optimal cutoff 1,282 mmHg%) and GLS (AUC = 0.938; 95% CI: 0.856–0.994; optimal cutoff −15.5%). Comparisons between AUCs were performed using DeLong's test for paired ROC curves. GWI and GCW showed good correlation with NT-proBNP levels (*r* = −0.567 and *r* = −0.552, respectively, both *p* < 0.001) in our cohort, similar to LVEF by the Simpson method (*r* = −0.594, *p* < 0.001). Myocardial work parameters may be promising markers of early LV systolic dysfunction in Chagas cardiomyopathy and may serve as useful prognostic markers for patients with CCC.

## Highlights

• Chagas disease is still a prevalent disease, and cardiac involvement represents an important cause of morbidity and mortality in endemic regions.
• Subclinical cardiac disease can be detected by two-dimensional longitudinal strain (2DS), but this technique can be influenced by afterload.
• Myocardial Work is a new technique that integrates non-invasive arterial blood pressure in the analysis of 2DS, but until today, we haven't had data about the use of this technique in different clinical stages of Chagas disease.
• Global work index and global constructive work had a good correlation with left ventricular (LV) ejection fraction and were the best parameters to detect global LV dysfunction in these patients, when compared with conventional parameters and NT-proBNP.
• There was a good correlation of GWI, GCW with NT-proBNP levels, better than global longitudinal strain and LVEF.

## Introduction

Chagas disease (CD) is a chronic infectious disease caused by a flagellated protozoan parasite, *Trypanosoma cruzi*, with 6,000,000–7,000,000 people estimated to be infected all over the world. It is still a prevalent cause of heart failure and cardiac death in endemic areas, concentrated in Central and South America, imposing a great burden on health systems in these countries, with marked socioeconomic impact (1). Due to migrations, prevalence is increasing in non-endemic areas, such as European countries and the United States, reinforcing the need to raise awareness about this deadly disease (2).

CD is still a poorly understood disease, and many inflammatory and immunologic pathophysiological mechanisms may play a role in its pathogenesis and clinical manifestations. Classically, a dormant and asymptomatic stage follows for a long period of time, with positive serology and without any gastrointestinal tract or cardiovascular abnormalities, called “indeterminate form.” After 20–30 years, approximately 30% of patients develop chronic cardiac Chagas disease, which may present with electrocardiographic (ECG) abnormalities, cardiomegaly on chest x-ray, left ventricular (LV) regional or global dysfunction on transthoracic echocardiogram (TTE), arrhythmias, overt clinical heart failure (HF), thromboembolic events, or even cardiac death (1, 2).

The prediction of which patients with indeterminate form will develop CCC in the future is still an unanswered question, and new markers of early disease may be beneficial for the timely institution of therapy. STE has been proven to be a very sensitive echocardiographic technique for the detection of subtle alterations in ventricular myocardial function in many cardiovascular diseases, including CD, and it can be reduced in patients with indeterminate form (3).

The evaluation of myocardial involvement in patients with CD was traditionally based on subjective evaluation of regional wall motion and global LV dysfunction, with accuracy limited by inter-observer variability and great dependence on image quality. Some studies have shown the value of GLS for predicting progression from indeterminate form to CCC (4, 5), while others have focused on its prognostic value in CCC patients. Early detection of myocardial alterations in CD patients by STE has been shown by Barbosa et al. (3) and García-Álvarez (6), studying a subset of CD patients in indeterminate form, demonstrating reduced regional longitudinal, radial, and circumferential deformation when compared with controls. Santos Junior et al. followed 81 patients with CCC (median follow-up = 18.2 months), finding that global longitudinal strain (GLS) was a strong predictor of adverse events (death, hospitalization for HF, heart transplantation), incremental to LVEF and *E*/*e*′ ratio (HR: 1.463, 95% CI: 1.130–1.894; *p* = 0.004). However, several studies have demonstrated that GLS is significantly influenced by loading conditions and chamber geometry (7).

**Figure 1:**
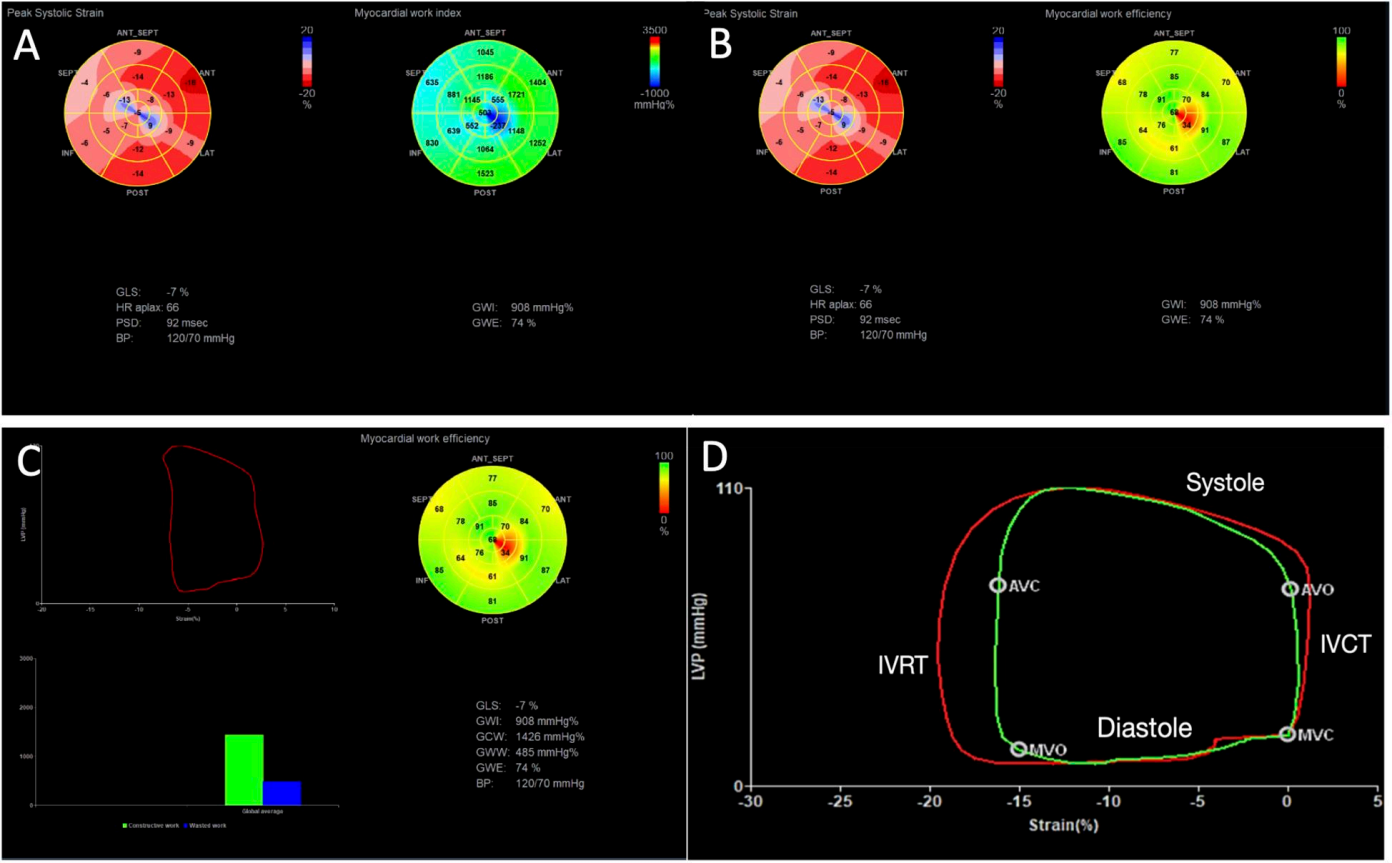
Example of myocardial work analysis in a patient with chronic cardiac Chagas disease and LV dysfunction (GLS = −7%). **(A)** We can visualize myocardial work index displayed in a bull’s-eye diagram (yellow arrow), with values for each myocardial segment. Global work index (GWI) = 908 mmHg%. **(B)** The median pressure–strain loop is displayed (red arrow), showing global constructive work (GCW) = 1,426 mmHg%, global wasted work (GWW) = 485 mmHg%, and global work efficiency (GWE) = 74%. **(C)** A bull’s-eye display shows values of GWE per myocardial segment. **(D)** A pressure–strain loop of a normal patient is displayed, showing in red the median work considering all segments, and in green the pressure–strain loop of a specific segment, showing its relation to the phases of the cardiac cycle. IVRT, isovolumetric relaxation time; IVCT, isovolumetric contraction time; MVO, mitral valve opening; MVC, mitral valve closure; AVO, aortic valve opening; AVC, aortic valve closure.

Myocardial work (MW) analyzed by echocardiography is a recently described technique, validated in 2012 (8), which uses an algorithm to integrate arterial blood pressure (ABP) (as a non-invasive measurement of afterload) to longitudinal strain measurements, generating pressure-deformation loops (Figure 1). MW-derived parameters have been proven to be a great tool for the evaluation of LV mechanical efficiency, with good correlation to myocardial oxygen consumption (9). There are also some clinical studies showing the prognostic value of MW in cardiomyopathies, such as cardiac amyloidosis (10) and cancer therapy-related cardiotoxicity (11), in patients with secondary mitral regurgitation (12), in patients with aortic stenosis (13), and in patients with advanced HF (14), highlighting the clinical utility of this new technique.

To the best of our knowledge, no studies have evaluated myocardial work in patients with CCC. In this study, we assessed myocardial work parameters across different stages of the disease (15) to explore their role in LV dysfunction and raise discussion about their potential prognostic value in patients with CCC.

## Methods

### Study population

This was a single-center, cross-sectional observational study. Data were retrospectively collected from a CD cohort. Consecutive patients from a Chagas cohort of a tertiary hospital were included from November 2021 to March 2023. The diagnosis of CD was established based on serological confirmation using two different methodologies [enzyme-linked immunosorbent assay (ELISA) and indirect immunofluorescence assay]. Exclusion criteria included the absence of adequate echocardiographic windows for performing a complete STE analysis or the presence of other significant cardiomyopathies not attributable to Chagas disease. All patients were submitted to a clinical anamnesis, ECG, NT-proBNP, and a comprehensive TTE.

Sample size was based on a paired comparison of detection proportions (GLS vs. myocardial work) using McNemar's test. Based on prior Chagas cohorts reporting ≈60% abnormal GLS (16), we powered the study to detect an absolute 20-percentage-point increase in detection by myocardial work (two-sided *α* = 0.05, 80% power), yielding *N* ≈ 48.

### Endpoints

• The primary endpoint was to evaluate MW in patients with CCC compared with conventional echocardiographic parameters.
• The secondary endpoints were to correlate MW parameters with NT-proBNP and the accuracy of these parameters to detect LV systolic dysfunction using LVEF by 2D Simpson as reference.

### Definitions of chronic Chagas cardiomyopathy

Electrocardiographic changes considered as defining of CCC were complete right bundle branch block (with or without left anterior fascicular block), second-degree or complete atrioventricular block, severe bradycardia (heart rate <40 bpm), sinus node dysfunction, primary T-wave abnormalities, inactive electrical zones, polymorphic premature ventricular complexes, non-sustained ventricular tachycardia, atrial fibrillation (AF), and left bundle branch block.

**Figure 2:**
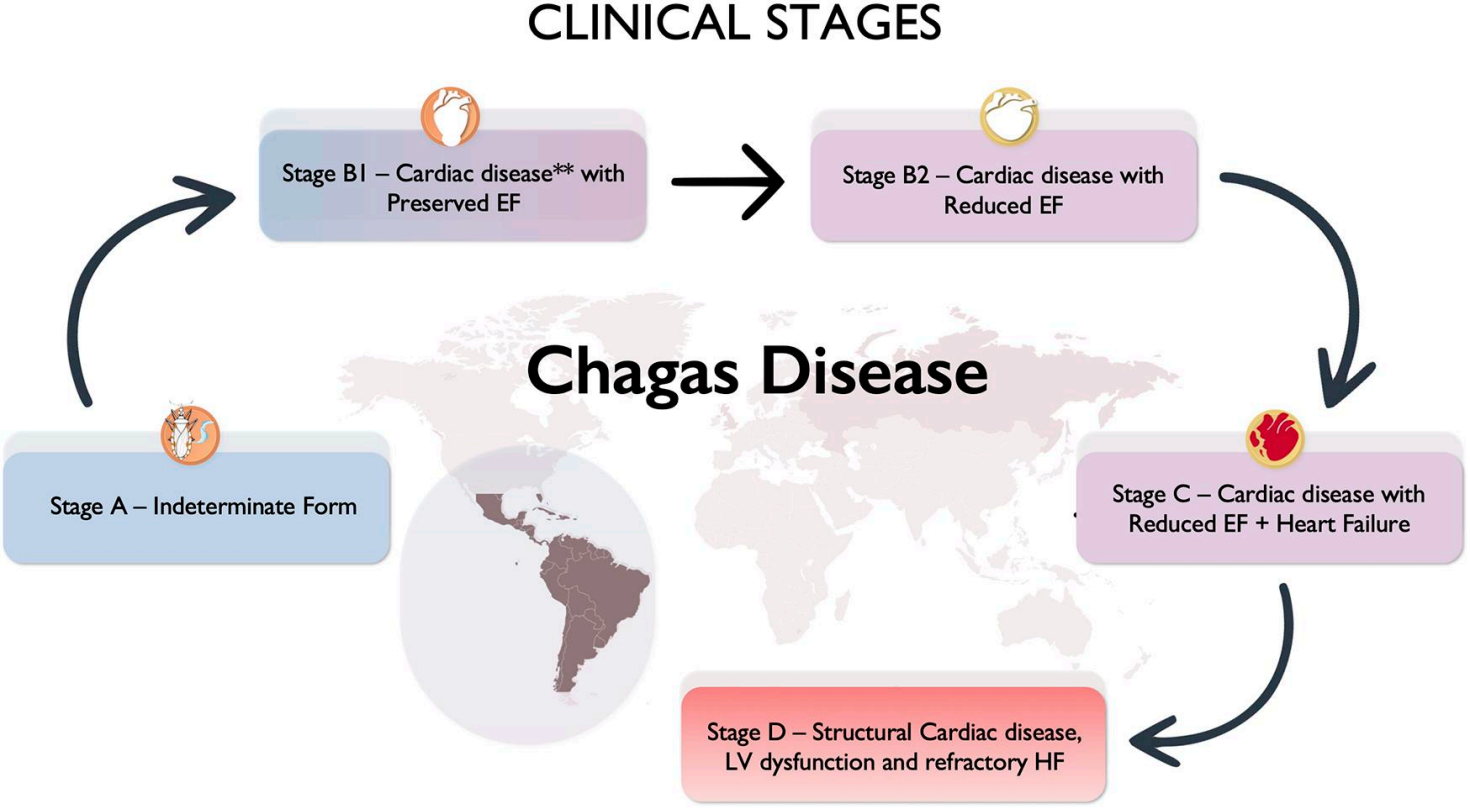
Clinical stages of chronic cardiac Chagas disease. **Cardiac disease: electrocardiogram—alterations such as right bundle block, left anterior fascicular block, left bundle block, atrioventricular complete block. Echocardiogram—wall motion abnormalities, left ventricular (LV) aneurysm, LV global dysfunction.

Patients were divided into progressive clinical stages using guidelines definitions (15), considering the presence of clinical (HF symptoms), electrocardiographic, and echocardiographic findings. We adapted and defined the LV ejection fraction (EF) threshold of 55% for LV systolic dysfunction (Figure 2), in summary:

1) Stage A (indeterminate form): no structural cardiac disease (normal ECG and Echo)—asymptomatic
2) Stage B1, structural cardiac disease: altered ECG and/or regional wall motion abnormalities. Preserved global systolic ventricular function (normal EF). No HF symptoms
3) Stage B2, structural cardiac disease: altered ECG and/or regional wall motion abnormalities. Reduced global systolic ventricular function (EF < 55%). No HF symptoms
4) Stage C, structural cardiac disease: global systolic ventricular dysfunction, with previous or current symptoms of HF
5) Stage D, structural cardiac disease: LV dysfunction and refractory HF

This project conforms to standards currently applied by the Brazilian National Committee for Research Ethics (CAE No: 47563415.9.0000.5272), and all subjects gave written informed consent before participation.

## Echocardiography

All patients were submitted to a comprehensive Echo, using standard views, with the patient in the left lateral decubitus position, using a commercially available ultrasound machine (Vivid E95, GE HealthCare, Horten, Norway). Non-invasive systemic ABP was obtained in this same position for the calculation of myocardial work parameters (17). Conventional echocardiographic images and cine loops of all patients were obtained by a single experienced examiner using an M5Sc transducer. All the measurements necessary to evaluate chambers’ dimensions, LV and right ventricular (RV) systolic function, parameters for quantification of diastolic function, grading severity of valvular lesions, and estimation of pulmonary artery systolic pressure (PASP) were obtained and analyzed in accordance with previously published guidelines (18). Three consecutive beats were stored for each view in patients with sinus rhythm and five beats for patients in atrial fibrillation. Two-dimensional LV volumes were determined using the modified Simpson rule with images obtained from apical four- and two-chamber views.

## Two-dimensional speckle-tracking echocardiography

For STE and myocardial work analysis, digital loops focusing on the LV were obtained from apical four-chamber, two-chamber, and three-chamber views. Three cardiac cycles were acquired for each view at a frame rate of 40–80 frames/s for patients in sinus rhythm and five consecutive cycles in patients with AF. The data were exported at the end of the exam to a dedicated workstation (EchoPAC 204, GE Vingmed, Horten, Norway) for further offline analysis.

An initial analysis was performed directly on the ultrasound machine to check if the image quality of the loops was good enough to permit adequate tracking of the acoustic markers (speckles) of the myocardium during the entire cardiac cycle. STE analysis was initially performed fully automated by the system, and manual editing was used to adjust the region of interest (ROI) to be aligned to the endocardial borders and fit to the thickness of the LV wall (ROI width). As the discretion of the sonographer, if tracking was poor, the operator could repeat the imaging or readjust technical settings. The ROI created by the software encompasses basal, mid, and apical segments of the LV, dividing it into six segments per view. Longitudinal peak strain values were measured for each segment using the automated function imaging (AFI) technique, and the 2D LV GLS was calculated by averaging the values from the six segments of each view.

Left atrial strain and RV longitudinal free wall strain were also obtained and analyzed, with technical settings in accordance with previous publications (19, 20).

## Myocardial work

Myocardial work assessment was performed with a vendor-specific algorithm (GE HealthCare, Pewaukee, WI, United States) using GLS (AFI), integrating afterload by generating a pressure–strain curve using non-invasive arterial blood pressure, obtained from the arm with a sphygmomanometer. Mitral valve (MV) and aortic valve (AV) opening and closure times were assessed automatically by the machine or manually for the definition of isovolumic and ejection phases, enabling the measurement of different work parameters in the pressure–strain loop (Figure 1).

## Myocardial work parameters

Myocardial parameters that can be obtained by TTE are as follows:

1. Global work index (GWI) = average myocardial work based on the pressure–strain loop (mmHg%)
2. Global constructive work (GCW) = positive work performed by a segment in systole and negative work (segment lengthening) during isovolumic relaxation.
3. Global wasted work (GWW) = negative work (segment lengthening) during systole and positive work (segment shortening) during isovolumic relaxation.
4. Global work efficiency (GWE) = GCW/(GCW + GWW).

Normal ranges for these parameters were published based on data from the Copenhagen City Heart Study (CCHS) study (Appendix 1) (21).

## Assessment of reproducibility

Twenty randomly chosen individuals were selected for inter-observer variability analysis by a second experienced observer and for intra-observer variability. All readers were blinded to previous measurements. Inter-observer and intra-observer variability were assessed using the intra-class correlation coefficient and Bland–Altman analysis.

## Statistical analysis

For descriptive analyses, continuous variables were expressed as mean ± standard deviation when normally distributed or as median (interquartile range) when asymmetrically distributed, and categorical variables were expressed as absolute numbers (percentages). The distribution of continuous variables was assessed using the Shapiro–Wilk test. For comparison of quantitative variables between groups of patients, the variance (ANOVA) test or Kruskal–Wallis test was used, depending on normality assessed by the Shapiro–Wilk test. Correlations between continuous myocardial work parameters and LVEF were assessed using Pearson's or Spearman's coefficients, according to data distribution. Differences between correlation coefficients were tested using Fisher's *r*-to-*z* transformation. ROC curve analyses were performed to determine the ability of each parameter to detect LV dysfunction (LVEF <55%), with AUCs and optimal cutoff values derived from the Youden index. Comparisons between AUCs were carried out using DeLong's test. All tests were bicaudal, and a *p*-value of <0.05 was considered statistically significant. Statistical analyses were performed using Jamovi software (The Jamovi Project, Version 2.6, IL, USA) and SPSS V 31.0.0.0.

## Results

Fifty patients were included in the study [32 women (64%), mean age: 64 ± 9]. Fourteen patients from our cohort were not included because they didn't agree to sign the informed consent. There were no exclusions, since all patients had adequate echocardiographic windows for performing a complete STE analysis, and no other significant cardiomyopathies except for CCC.

Clinical background, such as comorbidities and medical therapy, is described in Table 1. Additional clinical data, echocardiographic features, and NT-proBNP levels of the studied population are described in Table 2.

**Table 1:** Comorbidities and medical therapy of the general population and according to clinical stage groups.

Variables	Total (*n* = 50)	Group A (*n* = 9)	Group B1 (*n* = 18)	Group B2 (*n* = 13)	Group C (*n* = 10)
Clinical variables					
Arterial hypertension	33 (66%)	7 (78%)	14 (78%)	9 (69%)	3 (30%)
Diabetes mellitus	17 (34%)	4 (44%)	9 (50%)	3 (23%)	1 (10%)
Implanted cardiac defibrillator	17 (34%)	0 (0%)	5 (28%)	7 (54%)	5 (50%)
Dyslipidemia	30 (60%)	7 (78%)	12 (67%)	6 (46%)	5 (50%)
Medications					
Digoxin	6 (12%)	0 (0%)	0 (0%)	1 (8%)	5 (50%)
Beta-blockers	30 (60%)	3 (33%)	9 (50%)	9 (69%)	9 (90%)
ACE inhibitors/ARB	34 (68%)	7 (78%)	15 (83%)	11 (95%)	9 (90%)
Furosemide	19 (38%)	1 (11%)	4 (22%)	4 (31%)	10 (100%)
Calcium channel blockers	5 (10%)	0 (0%)	4 (22%)	0 (0%)	1 (10%)
Aldosterone antagonist	11 (22%)	0 (0%)	3 (17%)	2 (15%)	6 (60%)

ARB, angiotensin receptor blockers; ACE, angiotensin-converting enzyme.

**Table 2:** Clinical variables, echocardiographic features, and NT-proBNP levels of the studied population.

Variables	Total (*n* = 50)	Group A (*n* = 9)	Group B1 (*n* = 18)	Group B2 (*n* = 13)	Group C (*n* = 10)	*p*-value (between all groups)
Clinical variables						
Age (year)	66.8 ± 10.5	63.8 ± 11.5	72.3 ± 9.4	65.4 ± 9.0	61.7 ± 10.3	*p* = 0.055
Body surface area (cm^2^)	1.73 ± 0.17	1.80 ± 0.18	1.65 ± 0.16	1.80 ± 0.15	1.71 ± 0.15	*p* = 0.066
Systolic ABP (mmHg)	127 ± 25.6	144 ± 28.8^d^	137 ± 21.6	120 ± 17.8	105 ± 20^a^	*p* = 0.002
Diastolic ABP (mmHg)	73.4 ± 12.4	80.9 ± 16.8	75.6 ± 9.3	72.2 ± 9.4	64.1 ± 11.7	*p* = 0.050
Heart rate (bpm)	65.8 ± 10.8	67.9 ± 12.3	67.1 ± 11.4	62.6 ± 9.9	65.5 ± 10.1	*p* = 0.647
Chamber diameters						
Left atrium (mm)	40.9 ± 8.1	36.9 ± 4.3	40.7 ± 8.6	39.3 ± 5.5	46.8 ± 10.2	*p* = 0.065
LVEDd (mm)	51 (45.3–59.3)	47.8 ± 4.6^c^^,^^d^	46.8 ± 3.6^c^^,^^d^	58.8 ± 9.5	66.1 ± 9.6^a^^,^^b^	*p* < 0.001
LVESd (mm)	35.0 (28.3–49.0)	28.6 ± 3.2^c^^,^^d^	29.4 ± 3.7^c^^,^^d^	47.8 ± 9.1^a^^,^^b^	57.8 ± 8.9^a^^,^^b^	*p* < 0.001
LV systolic function						
EF biplane Simpson (%)	55 (36.5–71.0)	71.6 ± 5.1^c^^,^^d^	68.8 ± 8.6^c^^,^^d^	39.7 ± 5.0^b^^,^^d^	29.4 ± 4.8^a^^,^^b^^,^^c^	*p* < 0.001
MAPSE lateral (cm)	1.15 ± 0.30	1.42 ± 0.20^c^^,^^d^	1.30 ± 0.19^c^^,^^d^	0.97 ± 0.30^a^^,^^b^	0.88 ± 0.17^a^^,^^b^	*p* < 0.001
MAPSE septal (cm)	1.01 ± 0.29	1.24 ± 0.21^c^^,^^d^	1.13 ± 0.17^c^^,^^d^	0.85 ± 0.31^a^^,^^b^	0.78 ± 0.21^a^^,^^b^	*p* < 0.001
LV diastolic function						
LAVi (mL/m^2^)	39.0 (29.4–52.6)	29.2 ± 5.7^c^^,^^d^	39.5 ± 14.2^d^	42.7 ± 14.7^d^	64.3 ± 18.0^a^^,^^b^^,^^c^	*p* < 0.001
LASr (%)	21.1 ± 9.1	29.1 ± 6.0^c^^,^^d^	24.2 ± 8.2^d^	18.3 ± 7.7^a^	11.9 ± 5.1^a^^,^^b^	*p* < 0.001
LAEF (%)	49.6 (32.2–60.2)	57.1 ± 5.1^c^^,^^d^	51.4 ± 17.2^d^	41.3 ± 17.3^a^	27.4 ± 12.4^a^^,^^b^	*p* < 0.001
*e*′ lateral (cm/s)	8.46 ± 2.87	10.2 ± 2.5^c^	9.2 ± 3.0^c^	6.5 ± 1.9	6.0 ± 2.7	*p* = 0.008
*e*′ septal (cm/s)	6.75 ± 2.23	8.8 ± 2.4^c^^,^^d^	7.2 ± 2.2^c^	5.5 ± 1.1^a^^,^^b^	5.4 ± 1.4^a^	*p* = 0.003
*E*/*A*	0.78 (0.72–1.33)	0.92 ± 0.24	1.16 ± 0.98	0.99 ± 0.70	1.99 ± 0.92	*p* = 0.216
*E*/*e*′	6.00 (4.96–7.76)	5.07 ± 1.24^d^	6.35 ± 2.45	7.12 ± 2.86	8.72 ± 2.28^a^	*p* < 0.001
PASP (mmHg)	26.5 (23–36)	26.7 ± 4.5	29.0 ± 6.7	26.1 ± 6.6	40.2 ± 14.1	*p* = 0.079
RV systolic function						
TAPSE (cm)	2.15 (1.92–2.30)	2.2 ± 0.3	2.3 ± 0.3^d^	2.0 ± 0.48	1.8 ± 0.5^b^	*p* = 0.037
RVSTD (cm/s)	12.0 (10.0–12.8)	12.9 ± 1.8^d^	12.3 ± 1.4^d^	10.8 ± 1.8	9.3 ± 2.7^a^^,^^b^	*p* = 0.006
Laboratory data						
NT-proBNP (pg/mL)	150 (72.3–893)	61 (53–76)^d^	97.0 (68.5–184)^d^	302 (86.0–688)^d^	2,815 (1,985–3,306)^a^^,^^b^^,^^c^	*p* < 0.001

*Post hoc* analysis between groups (Games–Howell).

ABP, arterial blood pressure; LVEDd, left ventricular end diastolic diameter; LVESd, left ventricular end systolic diameter; EF, ejection fraction; MAPSE, mitral annular plane systolic excursion; LAVi, left atrial volume indexed; LASr, left atrial strain reservoir; LAEF, left atrial emptying fraction; PASP, pulmonary artery systolic pressure; RV, right ventricular; TAPSE, tricuspid annular plane systolic excursion; RVSTD, right ventricular systolic tissue Doppler; NT-proBNP, N-terminal pro-B-type natriuretic peptide.

^a^ Statistically significant difference when compared with clinical Stage A group. *P* < 0.05.

^b^ Statistically significant difference when compared with clinical Stage B1 group. *p* < 0.05.

^c^ Statistically significant difference when compared with clinical Stage B2 group. *p* < 0.05.

^d^ Statistically significant difference when compared with clinical Stage C group. *p* < 0.05.

There were nine patients in Stage A (18%), 18 patients in Stage B1 (36%), 13 patients in Stage B2 (26%), and 10 patients in Stage C (20%). There were no patients included in Stage D in this study since all individuals were recruited from an outpatient setting.

Age, heart rate, and body surface area were not statistically different between groups, with lower arterial blood pressure in Group C, compared with those in other groups.

NT-proBNP levels were significantly higher in Group C, compared with those in other groups (*p* < 0.001). Despite a higher median in Group B2, it was not significantly different when compared with Groups B1 (*p* = 0.411) and A (*p* = 0.091).

### Chamber diameters, diastole, and RV function

LV diameters were higher in Groups B2 and C, compared with those in Groups A and B1. Left atrial (LA) volume indexed (LAVi) was progressively dilated from Groups B1 and B2, albeit significantly larger in Group C. There was progressive alteration of LA reservoir strain (LASr), LA EF, reduced values of mean *e*′ in Groups B2 and C, pointing toward progressive diastolic dysfunction and elevation of LA pressures, although *E*/*e*′ was only significantly higher in Group C. PASP was not significantly different among groups (*p* = 0.079), although a higher median was registered in Group C.

RV function parameters were higher in Groups A and B1, when compared with those in Gruops B2 and C, as illustrated by values of RV free wall longitudinal strain (RVFWS) in Groups A (−23.9 ± 2.9) and B1 (−24.9 ± 4.8) higher in absolute values than Groups B2 (−20.8 ± 4.6) and C (−20.2 ± 5.1) (*p* = 0.048).

### Longitudinal function: myocardial deformation and myocardial work

**Figure 3:**
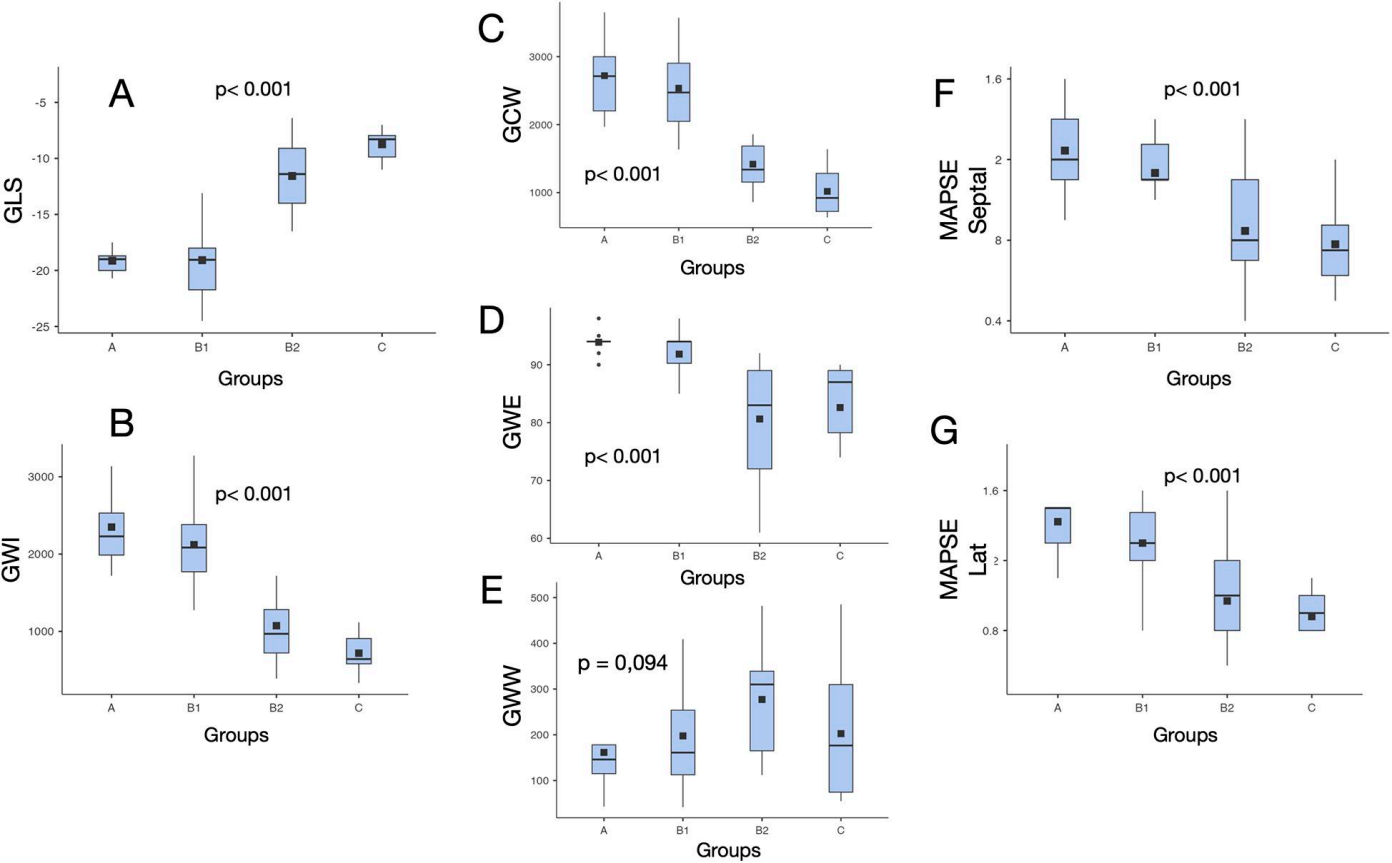
Values distribution of myocardial work indices and other echocardiographic systolic parameters in different clinical stages of chronic cardiac Chagas disease. *p = difference among all groups. GLS, left ventricular global longitudinal strain; GWI, global work index; GCW, global constructive work; MAPSE, mitral annular plane systolic excursion; GWE, global work efficiency; GWW, global wasted work; Lat, lateral.

GLS was significantly lower in Groups B2 (−11.6 ± 3.1%) and C (−8.7 ± 2.9%), when compared with that in Groups A (−19.5 ± 1.0%) and B1 (−19.1 ± 3.1%) (*p* < 0.001), but not significantly different from those in Groups B2 and C (*p* = 0.147). There was no significant difference between Groups A and B1 (*p* = 0.713) (Table 3, Figure 3).

**Table 3:** Two-dimensional longitudinal strain and myocardial work parameters of patients with Chagas disease divided into clinical stages.

Variables	Total (*n* = 50)	Group A (*n* = 9)	Group B1 (*n* = 18)	Group B2 (*n* = 13)	Group C (*n* = 10)	*p*-value (between all groups)
2D strain						
GLS LV AFI (%)	−16.0 (−19.1—10.5)	−19.5 ± 1.0	−19.1 ± 3.1	−11.6 ± 3.1	−8.7 ± 2.9	*p* < 0.001
Myocardial work						
GWI (mmHg%)	1,609 ± 804	2,349 ± 495	2,120 ± 536	1,073 ± 490	717 ± 257	*p* < 0.001
GCW (mmHg%)	1,975 ± 864	2,720 ± 613	2,537 ± 585	1,418 ± 473	1,018 ± 364	*p* < 0.001
GWW (mmHg%)	177 (114–306)	162 ± 81.6	198 ± 124	277 ± 116	203 ± 149	*p* = 0.094
GWE (%)	90.0 (83.5–94.0)	93.9 ± 2.1	91.8 ± 4.9	80.6 ± 10.6	82.6 ± 9.4	*p* < 0.001

Statistical significance: *p* < 0.005.

2D, two-dimensional; GLS, global longitudinal strain; LV, left ventricular; AFI, automated function imaging; GWI, global work index; GCW, global constructive work; GWW, global wasted work; GWE, global work efficiency.

GWI was significantly lower in Groups B2 (1,073 ± 490) and C (717 ± 257) compared with that in Groups A (2,349 ± 495) and B1 (2,120 ± 536) (*p* < 0.001), but not significantly different from those in Groups B2 and C (*p* = 0.146). There was no significant difference between Groups A and B1 (*p* = 0.693) (Table 3, Figure 3).

GCW was significantly lower in Groups B2 (1,418 ± 473) and C (1,018 ± 364) compared with that in Groups A (2,720 ± 613) and B1 (2,537 ± 585) (*p* < 0.001), but not significantly different from those in Groups B2 and C (*p* = 0.131). There was no significant difference between Groups A and B1 (*p* = 0.877) (Table 3, Figure 3).

GWE was significantly lower in Groups B2 (80.6 ± 10.6) and C (82.6 ± 9.4) compared with that in Groups A (93.9 ± 2.1) and B1 (91.8 ± 4.9) (*p* < 0.001), but not significantly different from those in Groups B2 and C (*p* = 0.964). There was no significant difference between Groups A and B1 (*p* = 0.442) (Table 3, Figure 3).

There was no statistically significant difference in GWW between groups.

### Correlation of parameters to LVEF and NT-proBNP

**Figure 4:**
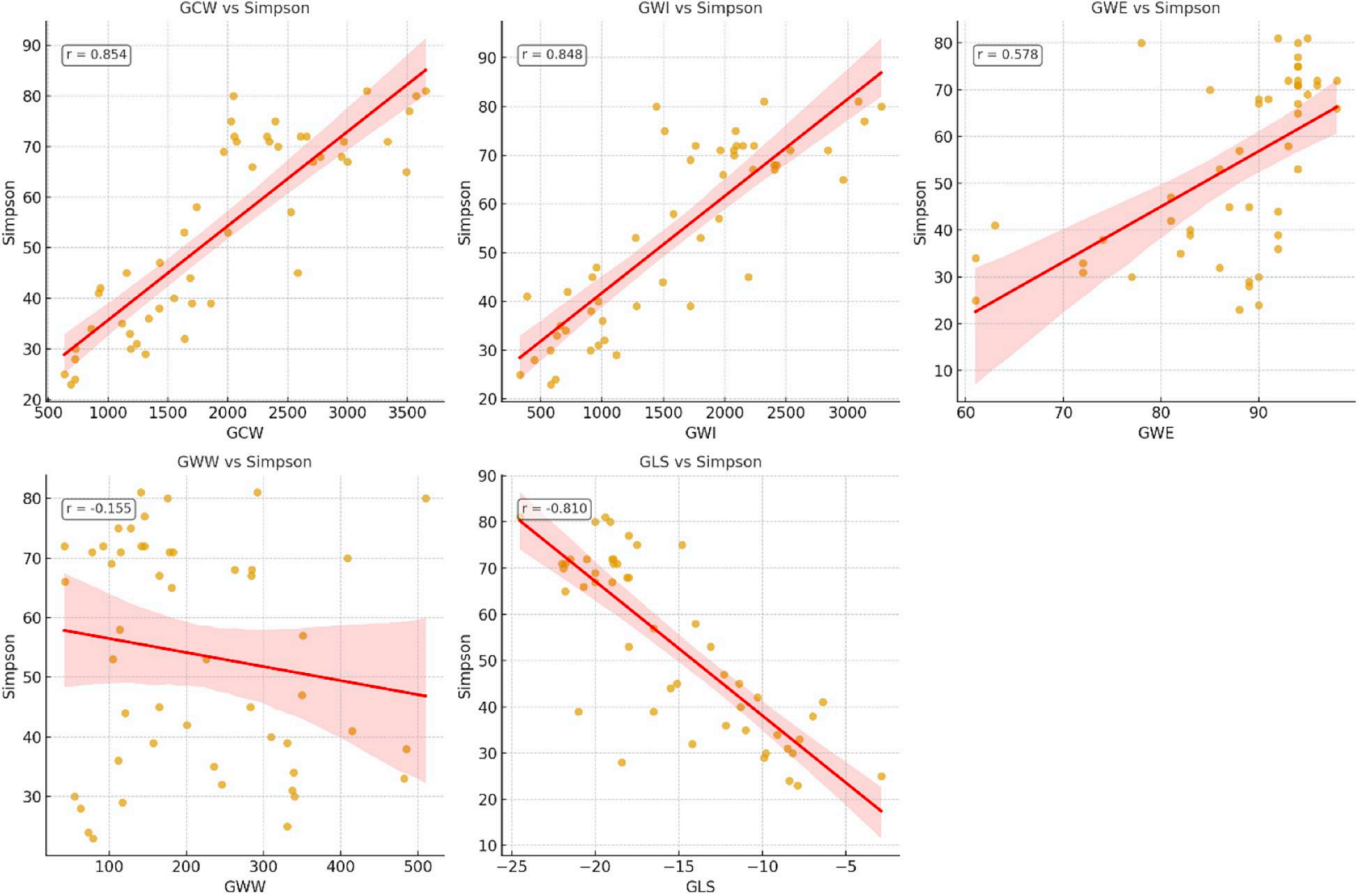
Correlation of echocardiographic parameters and left ventricular ejection fraction (LVEF) measured by biplanar Simpson. GCW, global constructive work; GWI, global work index; GWE, global work efficiency; GWW, global wasted work; GLS, global longitudinal strain.

Among the studied parameters, GCW showed the strongest correlation with LVEF (*r* = 0.854), followed by GWI (*r* = 0.848) and GLS (*r* = 0.810), while moderate correlations were observed for GWE (*r* = 0.578) and NT-proBNP (*r* = 0.634). Lateral mitral annular plane systolic excursion (MAPSE) demonstrated a moderate association (*r* = 0.596), whereas septal MAPSE exhibited a weaker correlation (*r* = 0.481). GWW had no significant correlation with LVEF (*r* = −0.155; *p* = 0.459) in our cohort. Pairwise comparisons of correlation coefficients using Steiger's *Z* test revealed that GCW had a significantly stronger correlation with LVEF than NT-proBNP (*p* < 0.001) and MAPSE (*p* < 0.01), but no significant difference was observed when compared with GLS or GWI (*p* > 0.05) (Table 4, Figure 4).

**Table 4:** Correlation of parameters with ejection fraction by biplane Simpson.

Parameter	*R*	*p*-value
GWI	0.848	*p* < 0.001
GCW	0.854	*p* < 0.001
GWW	−0.155	*p* = 0.459
GWE	0.578	*p* < 0.001
GLS	−0.810	*p* < 0.001
MAPSE lateral	0.596	*p* < 0.001
MAPSE septal	0.481	*p* < 0.001
NT-proBNP	−0.634	*p* < 0.001

Statistical significance: *p* < 0.005.

GLS, global longitudinal strain; GWI, global work index; GCW, global constructive work; GWW, global wasted work; GWE, global work efficiency; MAPSE, mitral annular plane systolic excursion; NT-proBNP, N-terminal pro-B-type natriuretic peptide.

**Figure 5:**
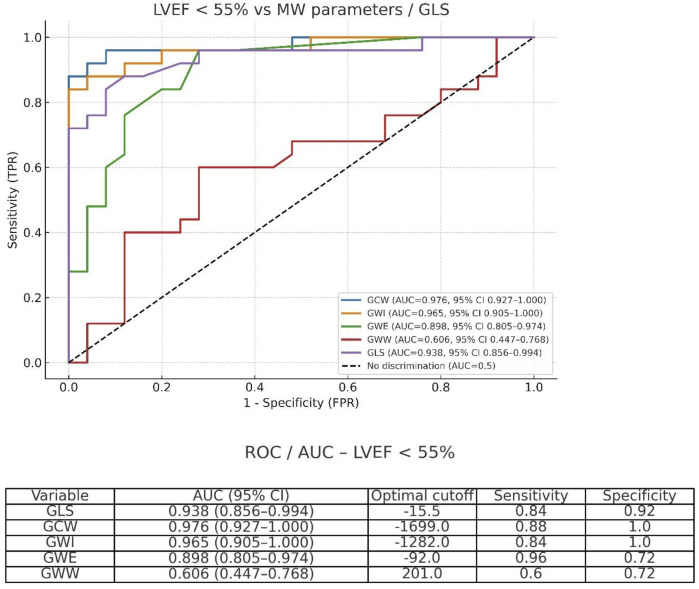
Receiver operating characteristic (ROC) curves for GCW, GWI, GWE, GWW, and GLS in detecting left ventricular dysfunction defined as LVEF <55%.

For the detection of LV global dysfunction as a categorical variable, defined as LVEF <55%, the best accuracy was observed for GCW (AUC = 0.976; 95% CI: 0.927–1.000; optimal cutoff 1,699 mmHg%), followed by GWI (AUC = 0.965; 95% CI: 0.905–1.000; optimal cutoff 1,282 mmHg%) and GLS (AUC = 0.938; 95% CI: 0.856–0.994; optimal cutoff −15.5%). Comparisons between AUCs were performed using DeLong's test for paired ROC curves (Figure 5).

GWI and GCW showed good correlation with NT-proBNP levels (*r* = −0.567 and *r* = −0.552, respectively, both *p* < 0.001) in our cohort, similar to LVEF by the Simpson method (*r* = −0.594, *p* < 0.001). GLS absolute values also correlated with NT-proBNP (*r* = 0.514, *p* < 0.001). In contrast, GWE demonstrated only a weak and non-significant association (*r* = −0.254, *p* = 0.075), while GWW showed no significant correlation (*r* = −0.043, *p* = 0.766). Considering diastolic parameters, LA strain had a great correlation with NT-proBNP levels (*r* = 0.69), better than *E*/*e*′ (*r* = 0.51, *p* < 0.001).

## Intra-observer and inter-observer variability analysis

Reproducibility analysis showed excellent concordance between repeated measurements for all MW parameters on the Bland–Altman analysis. GWI, GCW, GWE, and GWW showed a high intra-class correlation coefficient (range: intra-observer, 0.961–0.981; inter-observer, 0.960–0.978) with narrow confidence intervals (Table 5).

**Table 5:** Reproducibility analysis for retest measurements of myocardial work parameters.

Reproducibility analysis	ICC	CI (95%)
GWI intra-observer	0.965	0.928–0.990
GWI inter-observer	0.978	0.924–0.992
GCW intra-observer	0.981	0.948–0.993
GCW inter-observer	0.977	0.925–0.989
GWE intra-observer	0.966	0.933–0.986
GWE inter-observer	0.965	0.921–0.984
GWW intra-observer	0.961	0.930–0.985
GWW inter-observer	0.960	0.928–0.987

GWI, global work index; GCW, global constructive work; GWE, global work efficiency; GWW, global wasted work; ICC, intra-class correlation coefficient; CI, confidence interval.

## Discussion

This study evaluates MW parameters in patients with different clinical stages of cardiac involvement in chronic Chagas disease, and their correlation to LVEF and NT-proBNP levels. There have been insufficient clinical studies focused on early diagnosis and predicting evolution from indeterminate to determinate CCC. Some clinical scores are used for risk stratification in CCC, such as the Rassi score (22), which integrates demographic factors (male sex), HF symptoms (NYHA II–III), incidence of arrhythmias, cardiomegaly on chest x-ray, low voltage on ECG, and regional wall motion or LV dysfunction on TTE. All these echocardiographic parameters “classically” used are subjective, less sensitive, and potentially inaccurate, opening a great window for new echocardiographic quantitative parameters, as 2D strain-derived parameters or LV MW indices. GLS is correlated to myocardial fibrosis, determined non-invasively by late gadolinium enhancement on cardiac magnetic resonance (CMR) (23). Although some studies have shown the value of GLS (4), radial and circumferential strain (5) for predicting progression from indeterminate form to CCC, it is still a matter of intense debate, and new parameters are needed to understand the clinical progression of disease and prognostic assessment of these patients. Gomes et al. (23), studying indeterminate CD (Stage A) patients, showed lower longitudinal, circumferential, and radial strain values in patients with fibrosis detected on CMR, when compared with patients without fibrosis, despite similar EF, showing more accuracy of STE for detecting subclinical disease. Hotta et al. (24) showed that not only 2D LV GLS but also RV GLS, 3D LV GLS, 2D LV global circumferential strain, and 3D LV area strain were strong predictors of 60-month outcomes (hospitalization for HF, complex ventricular arrhythmias, heart transplant, and all-cause death) in patients with CD. Analysis of myocardial work, with integration of non-invasive ABP to GLS, creating a strain–pressure loop, provides less load-dependent data, which may allow us to obtain more accurate and reliable measurements. For instance, studies have shown a good correlation of MW to myocardial fibrosis in ischemic heart disease (25) and hypertrophic cardiomyopathy (26). A study by Hedwig et al. (27) found a strong correlation between GWI and NT-proBNP, cardiopulmonary exercise test, and LVEF, well-known markers of prognosis in patients with HF. Wang et al. (28) followed a cohort of 508 HF patients with EF <40% during 1 year and showed the independent prognostic value of GWI for the prediction of HF hospitalizations and all-cause mortality. Patients with GWI <750 mmHg% had a higher risk of all-cause death and HF hospitalization (HR: 3.33, 95% CI: 2.31–4.80) than patients with GWI >750 mmHg%.

In our study, GLS, GWI, GCW, and GWE were similar across Groups A and B1, with values within normal ranges, according to published reference data (29–31). GLS, GWI, and GCW were progressively reduced in Stage B2 and C groups, showing the loss of myocardial contractility and work in patients with LV global dysfunction and overt heart failure. GWI and GCW are independent predictors of outcome in patients with advanced heart failure (27) (death, LV assist device, and heart transplantation) and may be promising markers of disease progression in patients with CCC. GWE values were not different between Groups B2 and C (*p* = 0.964), although they were significantly reduced in both groups when compared with Groups A and B1. There was no significant difference in GWW between all groups, which could be explained by progressive loss of overall myocardial work (GWI) in patients with progressive cardiac disease, with less amount of work to be lost in an ineffective strain–pressure loop.

## Correlation of parameters with LVEF

In our study, GCW was the best parameter in correlation with LVEF (*r* = 0.854), followed by GWI (*r* = 0.848) and GLS (*r* = 0.810), showing the strength of these variables for the evaluation of LV systolic function in this population. In the study of Zhu et al. (32), studying 150 patients with coronary artery disease, a reduction of GWI and GCW was observed in patients with HF and reduced EF, while normal values were observed in patients with normal EF, or even an increase in a subset of patients with associated hypertension. Tomoaia et al. (33) showed in a group of 49 patients after acute myocardial infarction with heart failure and LVEF >40% a strong correlation between GWI and EF (*r* = 0.69) and only a moderate correlation with NT-proBNP. Frisan et al. (34) prospectively studied 119 patients with acute ST-elevation myocardial infarction, showing higher levels of GCW and GWI and lower GWW in patients with preserved (EF ≥ 50%) or mildly reduced EF (41%–49%), when compared with patients with reduced EF (≤40%), finding that GCW was the strongest predictor of adverse outcomes in the preserved LVEF group (AUC = 0.730, *p* = 0.035), while GWW demonstrated robust predictive performance in the reduced LVEF group (AUC = 0.787, *p* = 0.001).

GWE, MAPSE lateral, MAPSE medial, and NT-proBNP had only modest correlations with LVEF in our study. HF with preserved ejection fraction (HFpEF) can cause elevation of NT-proBNP with normal LVEF, and it can explain these results in this group of patients. GWW didn't show significant correlation with EF in our study (*p* = 0.459), maybe due to the inclusion of patients from different clinical stages, where low values of GWW could represent a small percentage in a patient with normal GWI or a greater amount in a patient with severe LV dysfunction and limited total GWI.

## Accuracy of parameters to detect LV global systolic dysfunction (EF < 55%)

Analyzing the accuracy of echocardiographic parameters to detect LV global dysfunction, defined as LVEF <55%, using receiver operating characteristic (ROC) curves, the area under the curve (AUC) was 0.976 for GCW, 0.965 for GWI, and 0.938 for GLS. Pairwise comparisons using DeLong's test showed no significant differences between GCW and GWI (*p* = 0.246), while GLS showed a trend toward lower performance compared with GCW (*p* = 0.068). Although accuracy was only slightly superior when compared with GLS, GWI and GCW may have the potential advantage of less bias caused by alterations of afterload. Kosmala et al. (35), analyzing a subset of 44 asymptomatic patients with high risk of cardiotoxicity during oncologic treatment, showed that myocardial work was superior to GLS to define cardiotoxicity in serial assessments, especially in patients with evolutive alterations of blood pressure, whereas an increase in GWI and GCW, even with decreased GLS, indicates the impact of elevated afterload on LV performance, without actual myocardial impairment.

## Correlation of myocardial work with NT-proBNP

A few studies have explored the correlation of myocardial work parameters with NT-proBNP. In the study of Roger-Rollé et al. (36), they showed good correlation of GWI with NT-proBNP (*r* = −0.518) in patients with cardiac amyloidosis, and during a median follow-up of 11 months, GWI and GWE were among the best predictors of mortality (AUC GWI = 0.626, AUC GWE = 0.689). In another study, 126 patients were followed after admission for acute heart failure, looking at myocardial response after decongestive treatment. GWI, GCW, and GWE were correlated negatively with NT-proBNP and positively with LVEF and *e*′, with a significant improvement of these parameters from admission to discharge in patients with LVEF <40% (37). Hedwig et al. (38) showed in a group of 51 patients with heart failure that GWI had a good correlation with peak oxygen consumption (peak VO_2_) (*r* = 0.521; *P* < 0.001) and with NT-proBNP (*r* = 0.635; *P* < 0.001). Patients with a GWI of <500 mm Hg% had a significantly higher NT-proBNP [median: 2,415 pg/mL (IQR: 1,071–5,933)].

GWI and GCW showed good correlation with NT-proBNP levels (*r* = −0.567 and *r* = −0.552, respectively, both *p* < 0.001) in our cohort, similar to LVEF by the Simpson method (*r* = −0.594, *p* < 0.001). GLS absolute values also correlated with NT-proBNP (*r* = 0.514, *p* < 0.001). In contrast, GWE demonstrated only a weak and non-significant association (*r* = −0.254, *p* = 0.075), while GWW showed no significant correlation (*r* = −0.043, *p* = 0.766). Chen et al. (39) studied 116 consecutive patients with dilated cardiomyopathy and found that GWI and GCW were not only independent predictors but also provided incremental predictive values of MACE in multivariate Cox models during a mean follow-up of 5.1years. Considering diastolic parameters, LA strain had a great correlation with NT-proBNP levels in our study (*r* = 0.693, *P* < 0.001), better than *E*/*e*′ (*r* = 0.515, *p* < 0.001), highlighting the power of this relatively new diastolic parameter to predict diastolic dysfunction with elevated left atrial pressure levels.

In our cohort, GWI, GCW, and GWE had better correlation with LVEF and GLS than NT-proBNP levels, and this may be explained by the interaction of other additional factors beyond myocardial contractility and systolic performance, as myocardial stretch, diastolic function, and pressure dynamics (preload and afterload). This was reinforced in our study by the great correlation of LA strain with NT-proBNP (*r* = 0.69). Diastolic dysfunction may be an early finding of CCC, and the use of LA strain and *E*/*e*′ offers incremental value in early-stage detection.

## Clinical perspective: what is new?

We described for the first time values of MW parameters in different stages of CCC and analyzed their correlation to LVEF and tested their accuracy to detect LV global dysfunction in these patients. We also compared its correlation with NT-proBNP levels, an important marker of HF and prognosis in these patients.

- GWI and GCW had normal values in Groups A and B1 and were progressively lower in Groups B2 and C.
- GCW was the best parameter in correlation with LVEF, followed by GLS and GWI, showing the strength of these variables for the evaluation of LV systolic function.
- The most accurate parameters for the detection of LV global dysfunction were GCW and GWI, followed by GLS.
- NT-proBNP levels were significantly higher in Group C, compared with other groups (*p* < 0.001). Despite a higher median in Group B2, it was not significantly different when compared with Groups B1 (*p* = 0.411) and A (*p* = 0.091).
- GCW and GWI may be prognostic markers of chronic cardiac Chagas disease, with the best correlation with NT-proBNP levels than GLS and LVEF.

## Limitations

Our findings must be validated in other studies involving a larger number of subjects. The relatively small sample size (*n* = 50) and uneven distribution of patients across different disease stages with the absence of Stage D patients can restrict the accuracy of some metrics in this study, particularly those related to ROC-based cutoff values for detecting LV dysfunction, and additionally does not allow us to extrapolate these results to other populations. This was a clinical, uncontrolled study, which included consecutive patients from a cohort of Chagas disease from a tertiary hospital, reflecting the population of patients currently treated in our clinical practice.

## Conclusion

In patients with Chagas disease in different clinical stages of the disease, GWI and GCW were normal in Groups A and B1, with progressive reduction in Groups B2 and C. These parameters had a great correlation with LVEF and had the best accuracy to detect LV global dysfunction, higher than GLS, and may serve as a new marker of myocardial function in CD. Based on these findings, GWI and GCW might be useful tools for the follow-up of patients with CCC and may serve as prognostic markers for this disease, which has to be studied in a larger prospective study.

## Data Availability

The raw data supporting the conclusions of this article will be made available by the authors, without undue reservation.

## Appendix 1: Table with normal values for myocardial work parameters, based on CCHS study results.

**Table:**

Myocardial work parameters	Total	Women	Men
GWI (mmHg%)	2,118 ± 277	2,155 ± 275	2,062 ± 269
GCW (mmHg%)	2,262 ± 283	2,283 ± 286	2,229 ± 275
GWW (mmHg%)	64 (47–89)	68 (49–93)	60 (42–84)
GWE (mmHg%)	97.1 (96.0–97.9)	97.0 (95.9–97.9)	97.2 (96.0–98.0)

Extracted from: Olsen et al. (21).

GWI, global work index; GCW, global constructive work; GWE, global work efficiency; GWW, global wasted work.
